# Diagnostic accuracy of genetic markers and nucleic acid techniques for the detection of *Leptospira* in clinical samples: A meta-analysis

**DOI:** 10.1371/journal.pntd.0008074

**Published:** 2020-02-12

**Authors:** Jia-Yong Lam, Gary Kim-Kuan Low, Hui-Yee Chee

**Affiliations:** 1 Department of Medical Microbiology and Parasitology, Faculty of Medicine and Health Sciences, Universiti Putra Malaysia, Selangor, Malaysia; 2 Clinical Research Team, Rapid Response Revival Research Ltd, Riverwood, Sydney, Australia; University of California San Diego School of Medicine, UNITED STATES

## Abstract

**Background:**

Leptospirosis is often difficult to diagnose because of its nonspecific symptoms. The drawbacks of direct isolation and serological tests have led to the increased development of nucleic acid-based assays, which are more rapid and accurate. A meta-analysis was performed to evaluate the diagnostic accuracy of genetic markers for the detection of *Leptospira* in clinical samples.

**Methodology and principle findings:**

A literature search was performed in Scopus, PubMed, MEDLINE and non-indexed citations (via Ovid) by using suitable keyword combinations. Studies evaluating the performance of nucleic acid assays targeting leptospire genes in human or animal clinical samples against a reference test were included. Of the 1645 articles identified, 42 eligible studies involving 7414 samples were included in the analysis. The diagnostic performance of nucleic acid assays targeting the *rrs*, *lipL32*, *secY* and *flaB* genes was pooled and analyzed. Among the genetic markers analyzed, the *secY* gene showed the highest diagnostic accuracy measures, with a pooled sensitivity of 0.56 (95% CI: 0.50–0.63), a specificity of 0.98 (95% CI: 0.97–0.98), a diagnostic odds ratio of 46.16 (95% CI: 6.20–343.49), and an area under the curve of summary receiver operating characteristics curves of 0.94. Nevertheless, a high degree of heterogeneity was observed in this meta-analysis. Therefore, the present findings here should be interpreted with caution.

**Conclusion:**

The diagnostic accuracies of the studies examined for each genetic marker showed a significant heterogeneity. The *secY* gene exhibited higher diagnostic accuracy measures compared with other genetic markers, such as *lipL32*, *flaB*, and *rrs*, but the difference was not significant. Thus, these genetic markers had no significant difference in diagnostic accuracy for leptospirosis. Further research into these genetic markers is warranted.

## Author summary

Leptospirosis is a globally important zoonotic disease is caused by *Leptospira* spp. This disease is often difficult to diagnose because its clinical manifestations resemble those of other diseases, such as dengue and malaria. Leptospirosis is often misdiagnosed, leading to improper medical management of patients. Accurate and timely diagnosis of the disease is important because antibiotic therapy is the most effective during the early stage. Nucleic acid assays are superior to most currently available diagnostics because they provide a definitive diagnosis during the acute stage of the disease even before antibodies are detectable. Nevertheless, the choice of genetic markers for these assays remains perplexing. Hence, this study evaluated the diagnostic accuracy of these genetic markers by pooling and analyzing them simultaneously. Results revealed that nucleic acid assays targeting the *secY* gene of leptospires had better diagnostic accuracy with the three other genetic markers coming close with good diagnostic performance. With the heterogeneity observed, there is no clear cut answer as to which of these markers is the best for diagnosing leptospirosis. Nevertheless, the analyses in this study suggested that all four markers exhibited good diagnostic measures and are promising targets for the future development of nucleic acid-based diagnostics.

## Introduction

Leptospirosis is a worldwide zoonotic disease recognized as an important emerging infectious disease in the past few decades. This disease occurs in diverse epidemiological settings, especially in tropical or subtropical regions of the world but imparts the greatest burden on resource-limited populations [1]. Leptospirosis was estimated to cause a million cases and close to 60,000 deaths annually [2]. Leptospirosis affects risk groups that are exposed to animal reservoirs or contaminated environments but exerts a broader health impact on impoverished farmers from the tropical regions [3]. This disease has also emerged as a health threat in new settings due to the influence of globalization and climate change, where natural disasters and extreme weather events are now recognized to precipitate epidemics [4, 5].

This disease is caused by spirochetes belonging to the genus *Leptospira*, comprising of both saprophytic and pathogenic species. The clinical manifestations of human leptospirosis are diverse, ranging from mild, flu-like illness to a more severe form of the disease known as Weil’s syndrome, which is characterized by jaundice, acute renal and hepatic failure, pulmonary distress, and hemorrhage, which can lead to death. These symptoms are similar to those of other infectious diseases, such as dengue fever and malaria, often causing misdiagnosis. Early diagnosis of this disease is crucial because antibiotic therapy provides the greatest benefit and is the most efficacious when initiated early in the course of an illness [3].

Dark-field microscopy is a conventional method for leptospirosis diagnosis through direct microscopic observation of clinical specimens. However, the sensitivity of this method is low, and the result is affected by the timing of sample collection and the skill of laboratory personnel [1]. *Leptospira* can be isolated from clinical specimens through inoculation into an appropriate culture medium, but its application in the field is hampered by the long doubling time and the need for special media in addition to its low sensitivity [6]. Microscopic agglutination test (MAT) is the current reference standard serological diagnostic test in leptospirosis. However, MAT requires the maintenance of live leptospires. As a minimum, the panel of live leptospires should include all locally circulating serovars; otherwise, an incomplete panel could lead to a false negative result [7]. Therefore, although MAT is considered as the gold standard test for leptospirosis diagnosis, it is laborious and its requirement for a large panel of live *Leptospira* culture hinders its standardization [8]. Other serological tests also have been developed with the likes of ELISA, complement fixation, indirect hemagglutination, latex bead agglutination, and indirect immunofluorescence [1, 3, 9], but all have been hampered by their low sensitivities for the initial management of acute leptospirosis [8].

These drawbacks have led to the increased development and use of nucleic acid-based diagnostics, such as conventional and real-time polymerase chain reaction (PCR) and isothermal amplification methods, which feature high sensitivity [10]. The advantage of nucleic acid-based diagnostics lies in their ability to obtain a definitive diagnosis during the acute stage of the disease even before antibodies are detectable [8]. Hence, these methods tend to replace the serological methods in endemic zones. They are normally based on the detection of a certain gene present in *Leptospira*. Genes such as *rrs*, *secY*, *lipL32*, *flaB*, *lfb1*, *ligA*, and *ligB2* have all been used as targets of nucleic acid-based diagnosis [11–14] and have been detected from blood, urine, cerebrospinal fluid, and tissue samples [15].

However, little is known about the diagnostic accuracy of each genetic marker. In addition, most studies had a low number of samples, which limited the statistical power and scientific reliability of the results. This meta-analysis was conducted to pool and analyze simultaneously all studies that used nucleic acid techniques to detect *Leptospira* in clinical samples of humans and animals. This pooled analysis aimed to provide a precise estimation of the diagnostic accuracy of nucleic acid techniques to detect *Leptospira*.

## Methods

### Literature search

A systematic review of nucleic acid techniques in detecting *Leptospira* was conducted based on the principles recommended in the Preferred Reporting Items for Systematic Reviews and Meta-analyses (PRISMA) statements (S1 Checklist).

#### Data sources

Relevant studies were identified by systematic search of electronic databases Scopus, PubMed, MEDLINE (from 1946 until present; via Ovid). and non-indexed citations (via Ovid).

#### Search strategy

The search of relevant studies was carried out up to December 2018 by using subject headings and free text terms. The search was carried out with the keywords “(leptospirosis OR leptospira*) AND (human OR patient OR animal OR clinical) AND (sensitivity OR specificity OR “true positive” OR “true negative” OR “false positive” OR “false negative”)”.

### Inclusion criteria

Cross-sectional and cohort studies that assessed nucleic acid techniques for the detection of *Leptospira* in human or animal clinical samples against at least one reference test were included, regardless of publication year. Laboratory diagnoses of leptospirosis are usually based on several methods or a combination of these methods due to the temporal nature of the disease progression and the absence of a satisfactory universal reference test [8]. Studies with reference tests such as MAT, any PCR-based tests, isolation of leptospires through culture, or the detection of antibodies to the bacteria, were considered for inclusion. Studies must directly or indirectly provide at least four values, which are number of true positives (TPs), false positives (FPs), true negatives (TNs), and false negatives (FNs), to construct or reconstruct a two-by-two table. Only articles published in English were evaluated.

### Exclusion criteria

The relevance of each study was determined based on their types. Reviews that do not contain original data and proceedings that did not employ any peer-review process were excluded. In addition, letters, editorials, and case reports were excluded. The objectives and methods were assessed, and studies were excluded if (1) samples were not tested by at least one reference test; (2) they involved spiked samples; (3) they involved experimentally infected animals; (4) data to derive a two-by-two table were insufficient; and (5) multiple genes were targeted in the index test.

### Data abstraction

#### Study selection

The titles and abstracts of potentially relevant studies from the literature search were screened by one reviewer in accordance with the eligibility criteria and further confirmed by a second reviewer. After the exclusion of duplicated records, studies without abstract, and apparently irrelevant studies, the full-text articles of remaining studies were screened by two reviewers. Disagreements about study inclusion and exclusion were resolved between the reviewers by consensus.

#### Data extraction and quality assessment

Data were extracted primarily by one reviewer and cross-checked by a second reviewer. Data collected from eligible studies included the first author name, publication year, characteristics of study population, number of samples, type of samples, type of method used as index test and reference test, and number of TPs, FPs, TNs, and FNs. Any disagreements between the two reviewers were documented and resolved through discussion with a third reviewer.

### Data analysis

The extracted data were compiled in a summary table, and the numbers of TPs, FPs, TNs, and FNs were used to calculate the sensitivity and specificity in each study. The pooled sensitivity, specificity, positive likelihood ratio (PLR), negative likelihood ratio (NLR), and diagnostic odds ratio (DOR) were determined for each group. For each statistic, the corresponding 95% confidence intervals (95% CI) were also calculated. The DOR is a single measure of diagnostic test performance that describes the odds of having a positive result in participants with a positive reference test compared against the odds in those with a negative reference test [16]. The DORs were evaluated using the DerSimonian-Laird method (random effects model) [17]. Summary receiver operating characteristic (SROC) curves that show the relationship between sensitivity and false positives rate (1 –specificity) were constructed to summarize the results. The area under the curve (AUC) of the SROC was calculated and proposed as a means to assess diagnostic data in the context of meta-analysis [18].

Heterogeneity was assessed by using *I*^2^ statistics and was interpreted as follows: an *I*^2^ value of less than 50% indicates homogeneity among the studies in the analysis, whereas an *I*^2^ value of more than 50% represents substantial heterogeneity among the studies [19]. One of the causes for heterogeneity observed in the meta-analysis of diagnostics is the threshold effect, which occurs if the studies use different thresholds to define a positive test result. In the present analysis, the presence of the threshold effect was determined by calculating the Spearman correlation coefficient between the sensitivity and specificity of the included studies [20]. In the absence of the threshold effect, meta-regression and subgroup analyses were performed to explore the contribution of individual factors on the heterogeneity observed, where a *p* value of less than 0.05 indicates a contribution to heterogeneity. All statistical analyses were carried out using Meta-DiSc software (version 1.4) [21].

## Results

### Literature search and study characteristics

**Fig 1:**
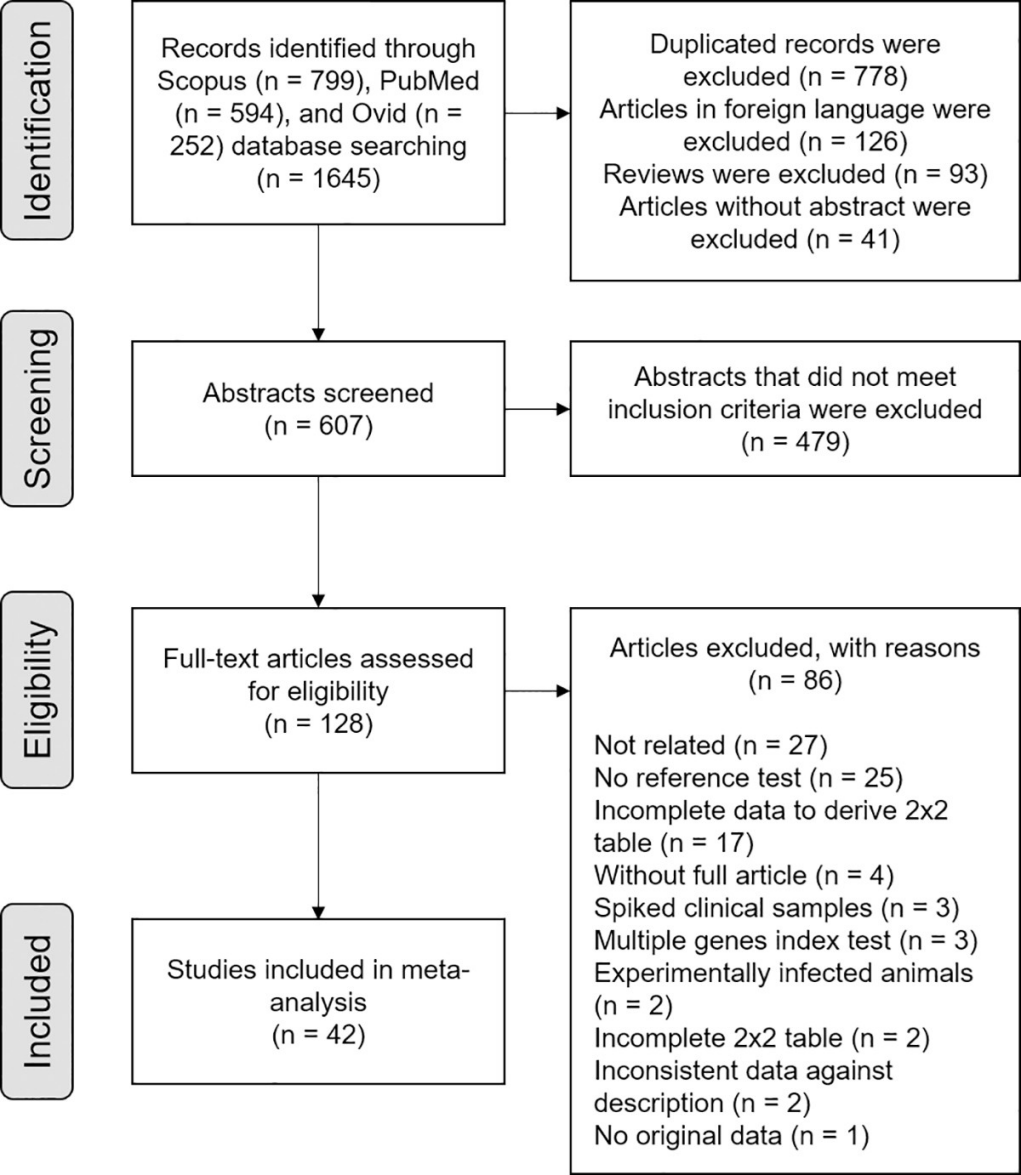
Flow chart of study selection process.

The study selection process is presented in Fig 1. The search for literature was completed in December 2018 and identified 1645 records, of which 128 full-text articles were retrieved to assess for eligibility. Of these full-text articles, 86 were excluded after further scrutiny. Forty-two studies involving 7414 samples met the inclusion criteria and were included in the meta-analysis. Detail and characteristics of each included study are presented in Table 1. The studies included were conducted in different countries and were published from 1992 to 2018. Some of the included studies used different methods; thus, the data were reported as separate independent studies [12, 22–32].

**Table 1:** Characteristics of studies included in the meta-analysis.

Study	Country	Study population	Number of samples	Sample type	Index test	Gene target	Reference test	TP	FP	FN	TN
[10]	Brazil	Suspected leptospirosis patients	478	Human—DNA from serum	Taqman qRT-PCR	*rrs*	MAT	3	32	30	413
[11]	Netherlands	Suspected leptospirosis patients	133	Human—DNA from serum or blood	SYBR Green qPCR	*secY*	Culture	15	8	0	110
[12]	Pacific Islands	Suspected leptospirosis patients	51	Human—DNA from serum	SYBR Green qPCR	*lfb1*	MAT	12	13	6	20
	Pacific Islands	Suspected leptospirosis patients	51	Human—DNA from serum	Nested PCR	*rrs*	MAT	12	13	6	20
[22]	Sri Lanka	Febrile patients	105	Human—DNA from blood	Taqman qPCR	*rrs*	MAT	9	1	40	55
	Sri Lanka	Febrile patients	105	Human—DNA from serum	Taqman qPCR	*rrs*	MAT	25	1	24	55
[23]	Brazil	Suspected leptospirosis patients	521	Human—DNA from serum	Conventional PCR	*rrs*	MAT	4	0	24	493
	Brazil	Suspected leptospirosis patients	521	Human—DNA from serum	Nested PCR	*rrs*	MAT	24	0	4	493
[24]	Brazil	Clinically confirmed leptospirosis patients, patients of other febrile diseases and healthy individuals	77	Human—DNA from blood or urine	Conventional PCR	LP1	MAT	11	0	22	44
	Brazil	Clinically confirmed leptospirosis patients, patients of other febrile diseases and healthy individuals	77	Human—DNA from blood or urine	Conventional PCR	*secY*	MAT	19	0	14	44
[25]	NR	Stray and household cats (healthy, non-vaccinated)	63	Animal—DNA from serum or urine	LAMP	*lipL32*	Taqman qPCR (*lipL32*)	22	0	2	39
	NR	Stray and household cats (healthy, non-vaccinated)	63	Animal—DNA from serum or urine	Nested PCR	*lipL32*	Taqman qPCR (*lipL32*)	17	3	7	36
	NR	Stray and household cats (healthy, non-vaccinated)	63	Animal—DNA from serum or urine	Conventional PCR	*rrs*	Taqman qPCR (*lipL32*)	1	0	23	39
	NR	Stray and household cats (healthy, non-vaccinated)	63	Animal—DNA from serum or urine	Conventional PCR	*secY*	Taqman qPCR (*lipL32*)	3	0	21	39
[26]	Philippines	Clinically confirmed leptospirosis patients	113	Human—DNA from urine pellet	SYBR Green qPCR	*flaB*	MAT	3	4	74	32
	Philippines	Clinically confirmed leptospirosis patients	113	Human—DNA from plasma or urine pellet	LAMP	*rrs*	MAT	2	3	75	33
[27]	Japan	Stray rats	18	Animal—Boiled urine sample	Nested PCR	*flaB*	Culture	6	0	6	6
	Japan	Stray rats	16	Animal—Urine pellet sample	Nested PCR	*flaB*	Culture	9	1	2	4
	Japan	Stray rats	18	Animal—Boiled urine sample	LAMP	*rrs*	Culture	11	2	1	4
	Japan	Stray rats	16	Animal—Urine pellet sample	LAMP	*rrs*	Culture	10	2	1	3
[28]	Thailand	Clinically confirmed leptospirosis patients	266	Human—DNA from blood	LAMP	*lipL41*	Culture, MAT	50	13	83	120
	Thailand	Clinically confirmed leptospirosis patients	266	Human—DNA from blood	LAMP	*rrs*	Culture, MAT	58	22	75	111
[29]	Brazil	Suspected leptospirosis patients	332	Human—DNA from serum	Taqman qPCR	*lipL32*	Culture, MAT	37	3	90	202
	Brazil	Suspected leptospirosis patients	332	Human—DNA from whole blood	Taqman qPCR	*lipL32*	Culture, MAT	77	12	50	193
[30]	Thailand	Febrile patients	266	Human—DNA from blood	Taqman qPCR	*lipL32*	Culture, MAT	57	9	76	124
	Thailand	Febrile patients	266	Human—DNA from blood	Taqman qPCR	*rrs*	Culture, MAT	74	14	59	119
[31]	Argentina	Clinically confirmed leptospirosis patients and non-cases	234	Human—DNA from serum or blood	Conventional PCR	*lipL32*	Culture, MAT	26	2	81	125
	Argentina	Clinically confirmed leptospirosis patients and non-cases	234	Human—DNA from serum or blood	Taqman qPCR	*lipL32*	Culture, MAT	47	9	60	118
[32]	Denmark	Suspected leptospirosis patients	51	Human—DNA from urine	Taqman qPCR	*lipL32*	MAT	3	1	0	47
	Denmark	Suspected leptospirosis patients	51	Human—DNA from urine	Taqman qPCR	*rrs*	MAT	3	1	0	47
[33]	Sri Lanka	Suspected leptospirosis patients	40	Human—DNA from serum	Taqman qPCR	*rrs*	MAT	5	5	11	19
[34]	NR	Suspected leptospirosis patients	63	Human—DNA from serum or blood	Recombinase polymerase amplification	*lipL32*	Culture	18	1	1	43
[35]	Brazil	Suspected leptospirosis patients	46	Human—RNA from blood	Taqman qRT-PCR	*rrs*	Culture, MAT, qPCR	14	0	8	24
[36]	Sri Lanka	Suspected leptospirosis patients	170	Human—DNA from blood	Nested PCR	*rrs*	MAT	7	7	54	102
[37]	Thailand	Febrile patients	418	Human—DNA from blood	Nested PCR	*rrs*	Culture	37	81	2	298
[38]	Barbados	Post-mortem samples	13	Human—DNA from organ	Conventional PCR	*secY*	Culture, MAT	2	0	6	5
[39]	USA	Random	34	Animal—Urine pellet sample	Conventional PCR	IS1500	MAT	23	3	7	1
[40]	Czech Republic	Suspected leptospirosis patients	852	Human—DNA from plasma, urine or CSF	Conventional PCR	*secY*	MAT	14	1	21	816
[41]	Sri Lanka	Suspected leptospirosis patients	95	Human—DNA from blood	SYBR Green qPCR	*secY*	MAT	44	3	21	27
[42]	Uruguay	Suspected leptospirosis patients	183	Human—DNA from serum	SYBR Green qPCR	*lipL32*	MAT	26	0	59	98
[43]	Turkey	Suspected leptospirosis patients and animals	133	Human and animal—DNA from serum	Nested PCR	*rrs*	MAT	90	2	0	41
[44]	NR	Suspected leptospirosis dogs	135	Animal—DNA from serum	Nested PCR	*rrs*	MAT	47	23	4	61
[45]	Thailand	Wild rodents	36	Animal—DNA from kidney	Taqman qPCR	*lipL32*	Conventional PCR (*gyrB*)	4	0	0	32
[46]	NR	Suspected leptospirosis patients and healthy controls	28	Human—DNA from urine, CSF or blood	Conventional PCR	*rrs*	MAT	4	0	2	22
[47]	Malaysia	Suspected leptospirosis patients	65	Human—DNA from blood	Taqman qPCR	*rrs*	Commercial GenoAmp qPCR leptospirosis kit	10	1	0	54
[48]	India	Suspected leptospirosis patients	207	Human—DNA from serum	Taqman qPCR	*lipL32*	MAT	84	10	77	36
[49]	India	Suspected leptospirosis patients	134	Human—NR	Conventional PCR	*secY*	MAT	34	4	1	95
[50]	NR	Suspected leptospirosis patients	42	Human—DNA from blood or urine	Conventional PCR	*flaB*	MAT	39	0	0	3
[51]	Brazil	Suspected leptospirosis patients	92	Human—DNA from serum	Conventional PCR	*secY*	MAT	17	13	30	32
[52]	India	Suspected leptospirosis patients	207	Human—DNA from blood	Nested PCR	*lipL32*	Culture	21	3	79	104
[53]	Nicaragua	Febrile patients	85	Human—DNA from blood	Taqman qPCR	*lipL32*	MAT	11	6	27	41
[54]	Brazil	Patients with meningeal abnormalities	39	Human—DNA from CSF	Conventional PCR	*rrs*	MAT	10	13	2	14
[55]	India	Suspected leptospirosis patients	100	Human—DNA from serum	Conventional PCR	*rrs*	MAT	2	4	16	78
[56]	India	Asymptomatic participants	196	Human—DNA from urine	Taqman qPCR	*rrs*	MAT	37	67	22	70
[57]	NR	Suspected leptospirosis patients	231	Human—DNA from serum or blood	Taqman qPCR	*rrs*	Culture	27	1	1	202
[58]	NR	Wild animals	220	Animal—DNA from serum	Taqman qPCR	*rrs*	MAT	0	1	14	205
[59]	Brazil	Suspected leptospirosis patients	55	Human—DNA from serum or plasma	Taqman qRT-PCR	*rrs*	MAT	6	47	0	2
[60]	Laos	Febrile patients	787	Human—DNA from blood or urine	Taqman qPCR	*rrs*	Culture, MAT	7	69	26	685

NR represents information not reported

### Accuracy of nucleic acid techniques targeting the *rrs* gene

**Fig 2:**
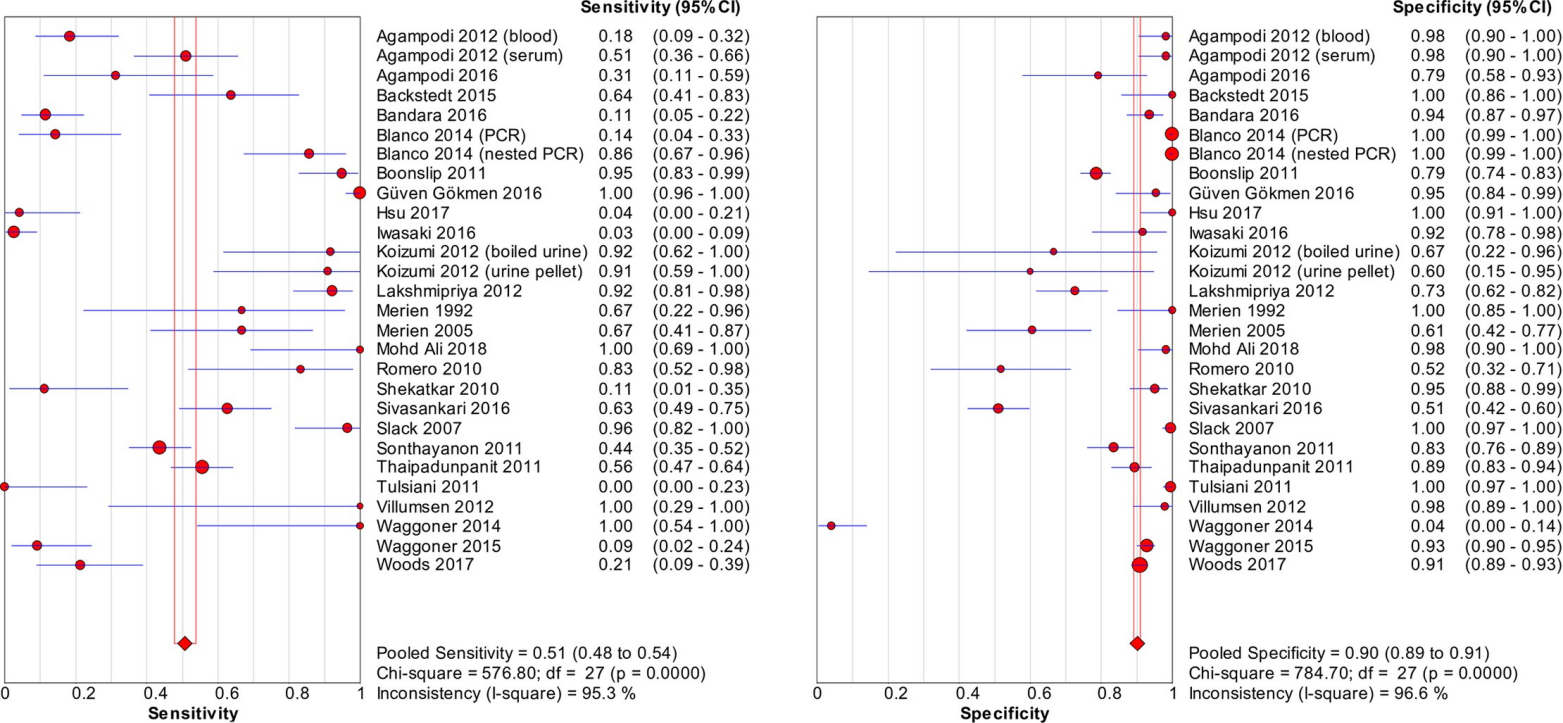
Forest plots of sensitivity and specificity of studies using *rrs* as the target gene for the detection of *Leptospira*.

**Fig 3:**
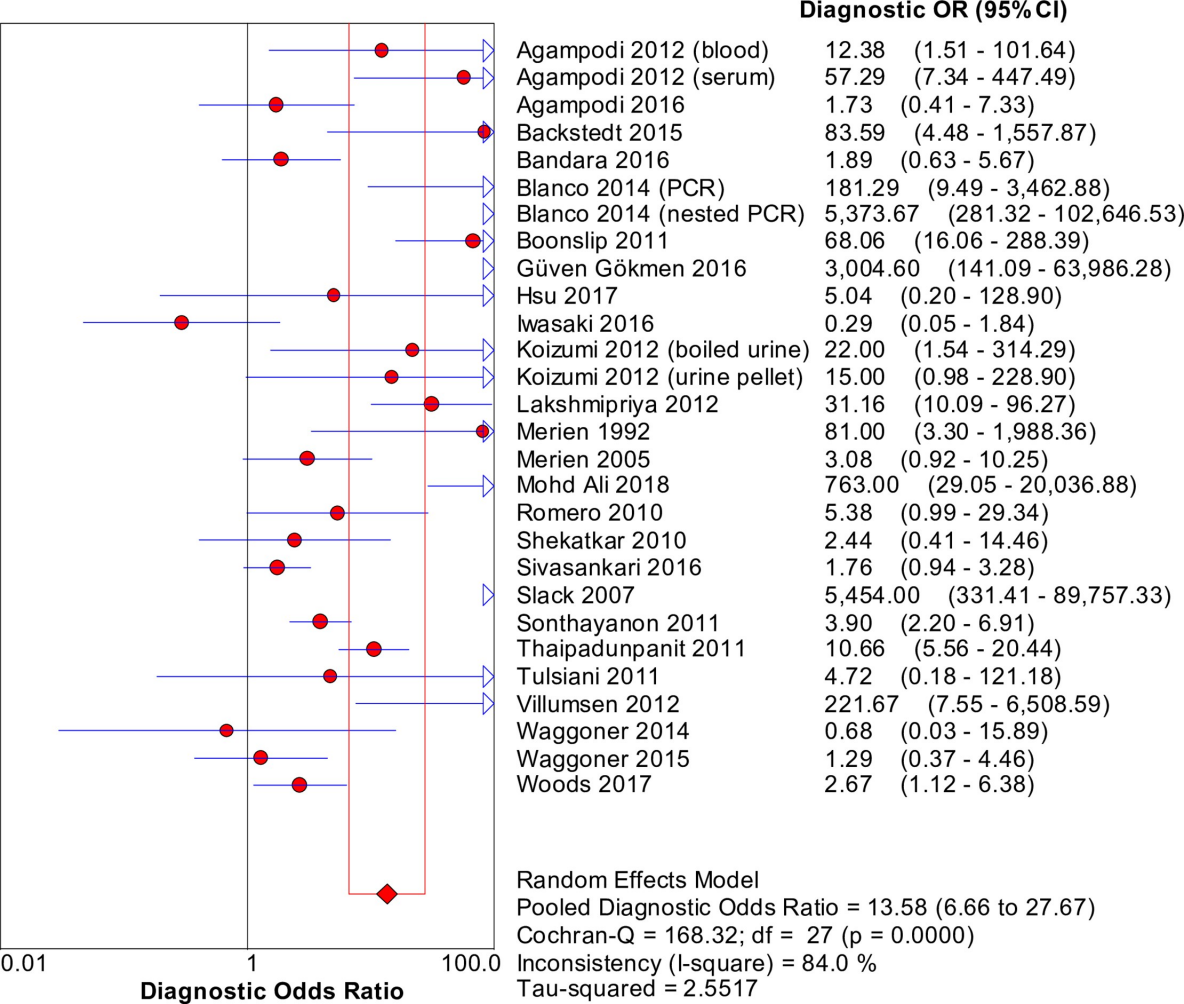
Forest plot of DOR of targeting the *rrs* gene in the detection of *Leptospira*.

**Fig 4:**
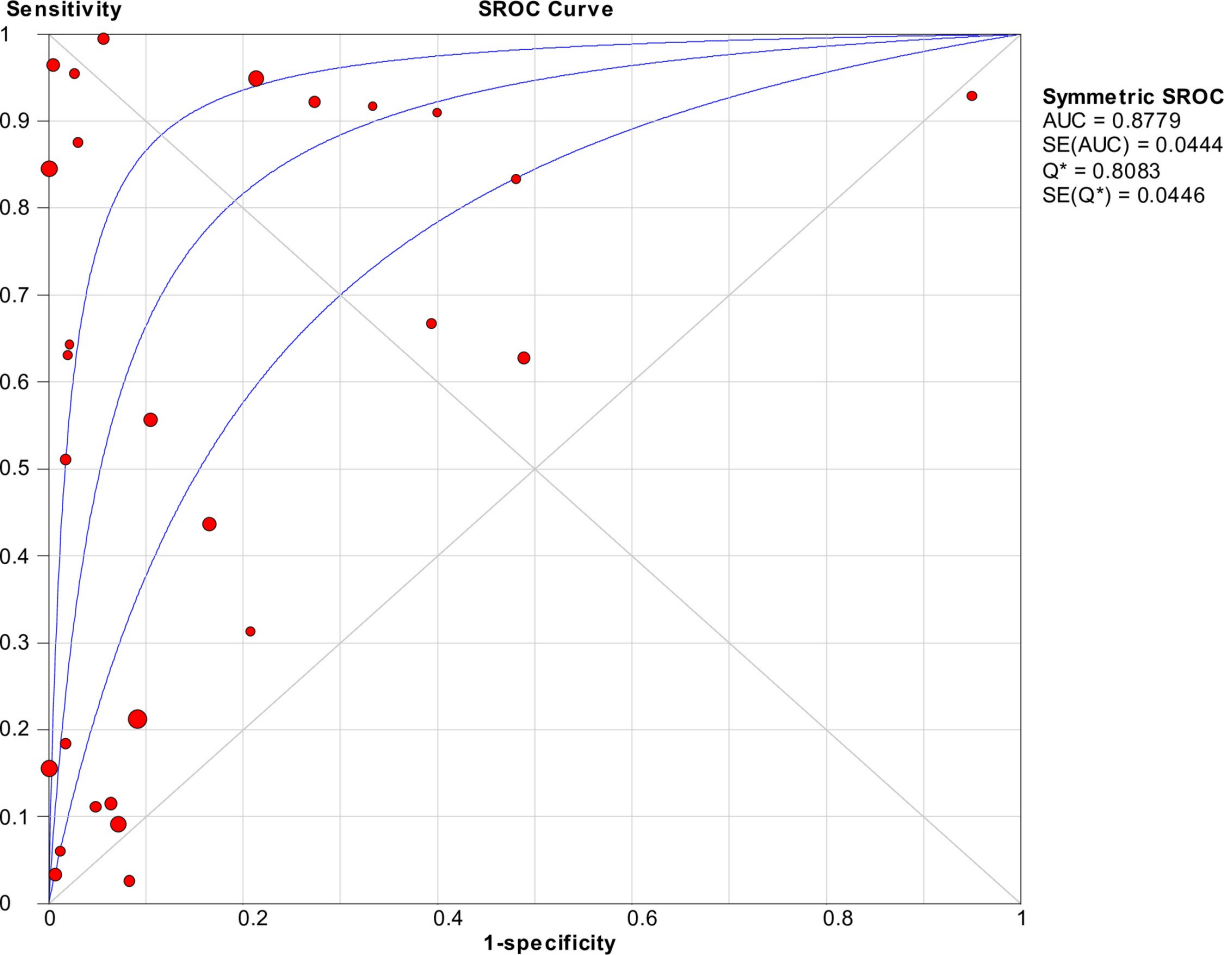
SROC curve of targeting the *rrs* gene in the detection of *Leptospira*.

The pooled sensitivity of techniques targeting the *rrs* gene was 0.51 (95% CI: 0.48–0.54), whereas the pooled specificity was 0.90 (95% CI: 0.89–0.91). Fig 2 shows the detailed forest plot of the sensitivities and specificities of the included studies. The pooled DOR was 13.58 (95% CI: 6.66–27.67), as shown in Fig 3. Fig 4 shows the SROC curve; the AUC and the pooled diagnostic accuracy (Q*) were 0.88 and 0.81, respectively. Significant heterogeneity was observed among the studies (sensitivity, *I*^2^ = 95.3%; specificity, *I*^2^ = 96.6%; DOR, *I*^2^ = 84.0%).

The Spearman correlation coefficient was calculated to be 0.273 with *p* = 0.160 (> 0.05), indicating that the significant heterogeneity was not due to the threshold effect. Hence meta-regression and subgroup analyses were performed to explore the overall heterogeneity and identify the source of heterogeneity, including the type of index test and type of sample (human or animal). The meta-regression analysis did not demonstrate that these two covariates contributed to the heterogeneity (*p* > 0.05 for both covariates). Subgroup analyses were performed on the basis of these covariates, and the results are shown in Table 2. Only one study dealt with human and animal samples; thus, analysis was not performed for this sample category.

**Table 2:** Meta-analysis results of studies targeting the *rrs* gene for the detection of *Leptospira*.

	Sensitivity (95% CI)	Specificity (95% CI)	PLR (95% CI)	NLR (95% CI)	DOR (95% CI)	AUC
**All studies**	0.51 (0.48–0.54)	0.90 (0.89–0.91)	4.36 (2.80–6.79)	0.56 (0.44–0.70)	13.58 (6.66–27.67)	0.88
**Subgroup (Type of index test)**						
**Conventional PCR**	0.24 (0.15–0.34)	0.97 (0.96–0.98)	7.09 (1.27–39.72)	0.86 (0.70–1.05)	11.61 (2.43–55.45)	0.78
**Nested PCR**	0.76 (0.70–0.80)	0.89 (0.87–0.91)	4.62 (2.32–9.21)	0.13 (0.01–2.20)	53.18 (6.83–413.92)	0.95
**qPCR**	0.50 (0.45–0.55)	0.90 (0.89–0.92)	8.14 (3.00–22.09)	0.62 (0.42–0.90)	17.21 (5.19–57.05)	0.92
**qRT-PCR**	0.38 (0.26–0.51)	0.85 (0.81–0.88)	2.82 (0.15–53.67)	0.68 (0.23–2.04)	3.80 (0.21–67.78)	0.68
**LAMP**	0.35 (0.29–0.41)	0.84 (0.78–0.89)	2.01 (1.04–3.91)	0.64 (0.34–1.18)	3.57 (0.70–18.23)	0.83
**Subgroup (Type of sample)**						
**Human samples**	0.44 (0.41–0.48)	0.90 (0.89–0.91)	4.45 (2.67–7.40)	0.61 (0.49–0.77)	11.09 (5.17–23.79)	0.83
**Animal samples**	0.62 (0.52–0.71)	0.92 (0.88–0.94)	3.22 (2.34–4.45)	0.39 (0.17–0.92)	20.90 (8.55–51.08)	0.89

### Accuracy of nucleic acid techniques targeting the *lipL32* gene

**Fig 5:**
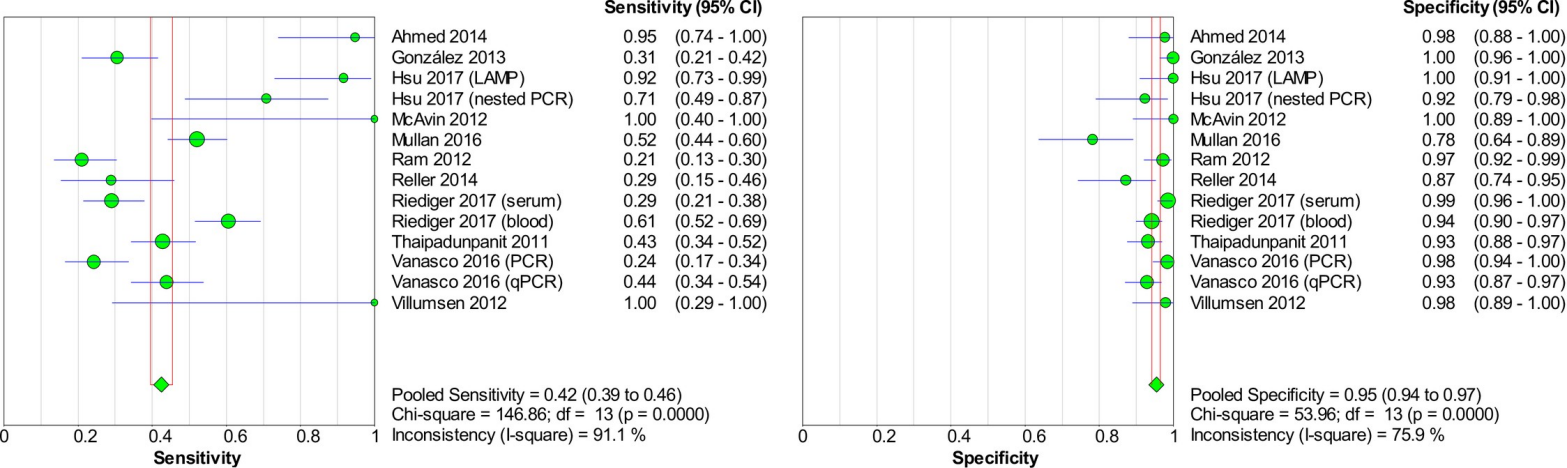
Forest plots of sensitivity and specificity of studies using *lipL32* as the target gene for the detection of *Leptospira*.

**Fig 6:**
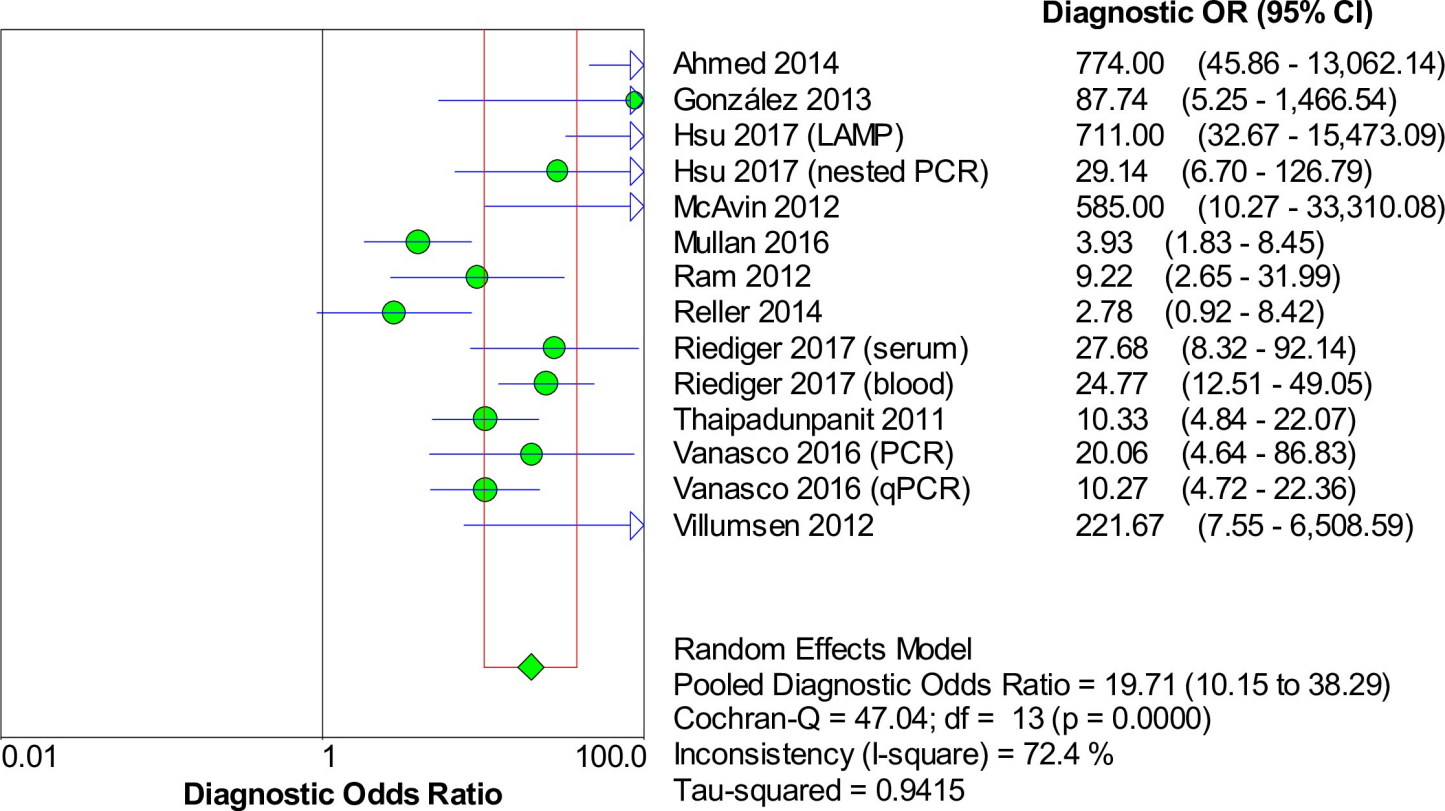
Forest plot of DOR of targeting the *lipL32* gene in the detection of *Leptospira*.

**Fig 7:**
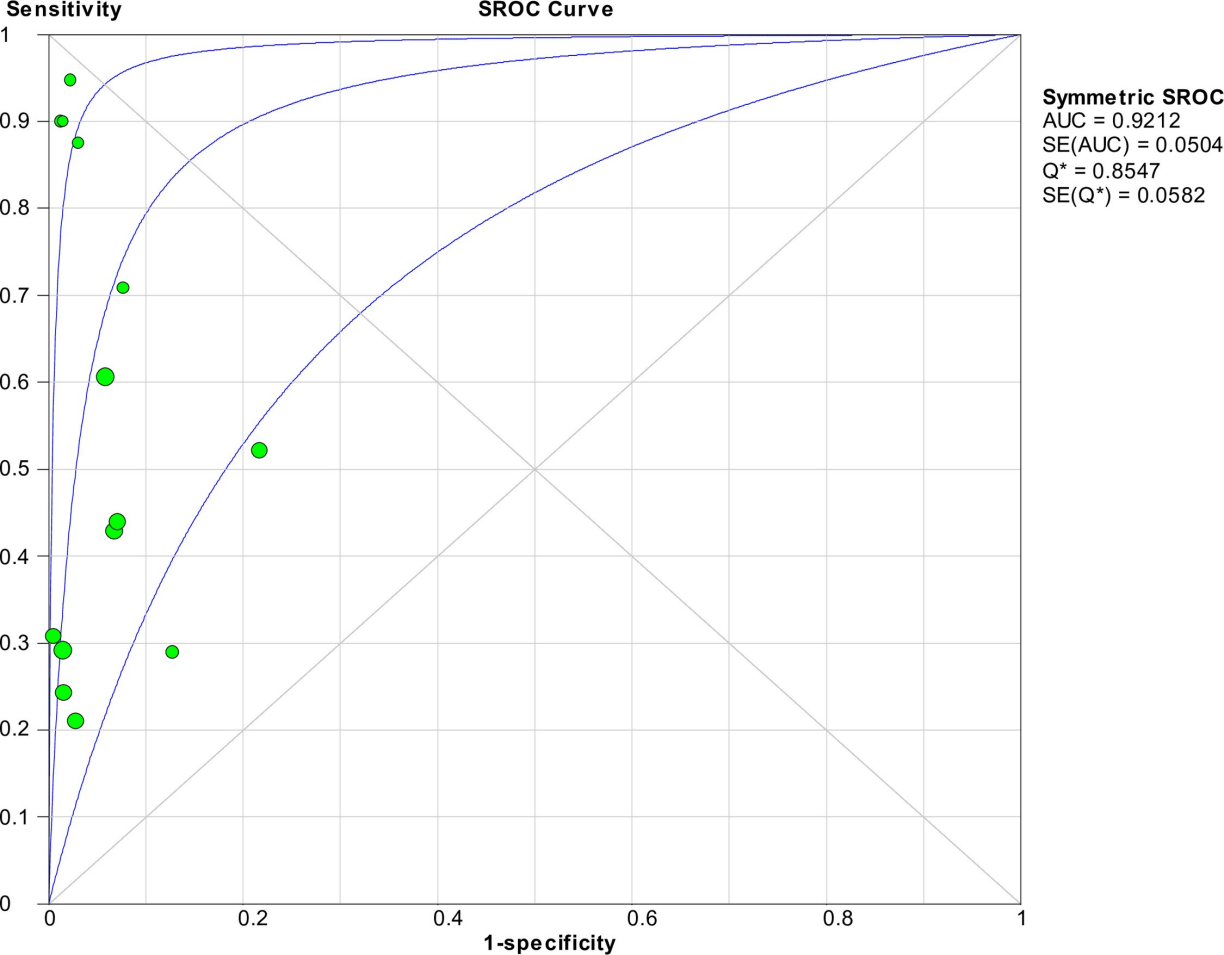
SROC curve of targeting the *lipL32* gene in the detection of *Leptospira*.

The pooled sensitivity and specificity of techniques using the *lipL32* gene as the target of detection were 0.42 (95% CI: 0.39–0.46) and 0.95 (95% CI: 0.94–0.97), respectively. The detailed forest plots of the sensitivities and specificities of the included studies are shown in Fig 5. The pooled DOR was 19.71 (95% CI: 10.15–38.29), as shown in Fig 6. The SROC curve is presented in Fig 7 with an AUC of 0.92 and Q* value of 0.85. Significant heterogeneity was observed among the studies (sensitivity, *I*^2^ = 91.1%; specificity, *I*^2^ = 75.9%; DOR, *I*^2^ = 72.4%).

The Spearman correlation coefficient was -0.077 with *p* = 0.794 (> 0.05), indicating that significant heterogeneity was not due to the threshold effect. Meta-regression and subgroup analyses were then performed to explore the heterogeneity and identify the source of heterogeneity. The meta-regression analysis did not demonstrate that the two covariates (type of index test and type of sample) contributed to the heterogeneity (*p* > 0.05 for both). Subgroup analyses were performed on the basis of these covariates, and the results are shown in Table 3. The AUC for the subgroup of index test (nested PCR) was not computed because of the low number of studies (n = 2). Analysis for the following index tests could not be performed because only one study was present in each category: conventional PCR, LAMP, and recombinase polymerase amplification.

**Table 3:** Meta-analysis results of studies targeting the *lipL32* gene for the detection of *Leptospira*.

	Sensitivity (95% CI)	Specificity (95% CI)	PLR (95% CI)	NLR (95% CI)	DOR (95% CI)	AUC
**All studies**	0.42 (0.39–0.46)	0.95 (0.94–0.97)	9.42 (5.64–15.75)	0.61 (0.51–0.71)	19.71 (10.15–38.29)	0.92
**Subgroup (Type of index test)**						
**Nested PCR**	0.31 (0.23–0.40)	0.96 (0.91–0.98)	8.35 (3.71–18.79)	0.52 (0.17–1.65)	15.31 (4.93–47.52)	NA
**qPCR**	0.44 (0.41–0.48)	0.95 (0.93–0.96)	7.83 (4.21–14.56)	0.62 (0.54–0.72)	13.64 (6.52–28.54)	0.75
**Subgroup (Type of sample)**						
**Human samples**	0.40 (0.37–0.44)	0.95 (0.94–0.96)	8.33 (4.82–14.37)	0.65 (0.57–0.75)	14.65 (7.57–28.32)	0.86

### Accuracy of nucleic acid techniques targeting the *secY* gene

**Fig 8:**
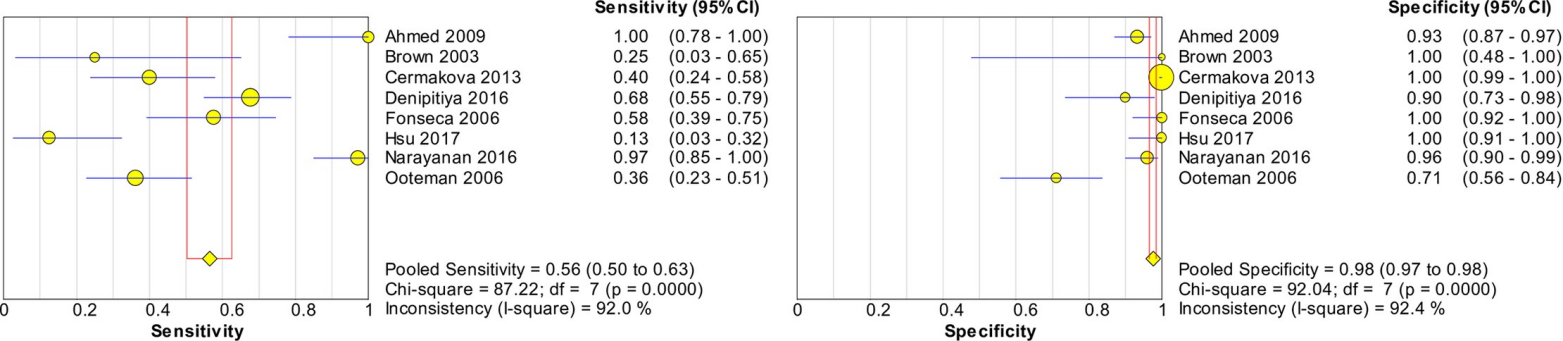
Forest plots of sensitivity and specificity of studies using *secY* as the target gene for the detection of *Leptospira*.

**Fig 9:**
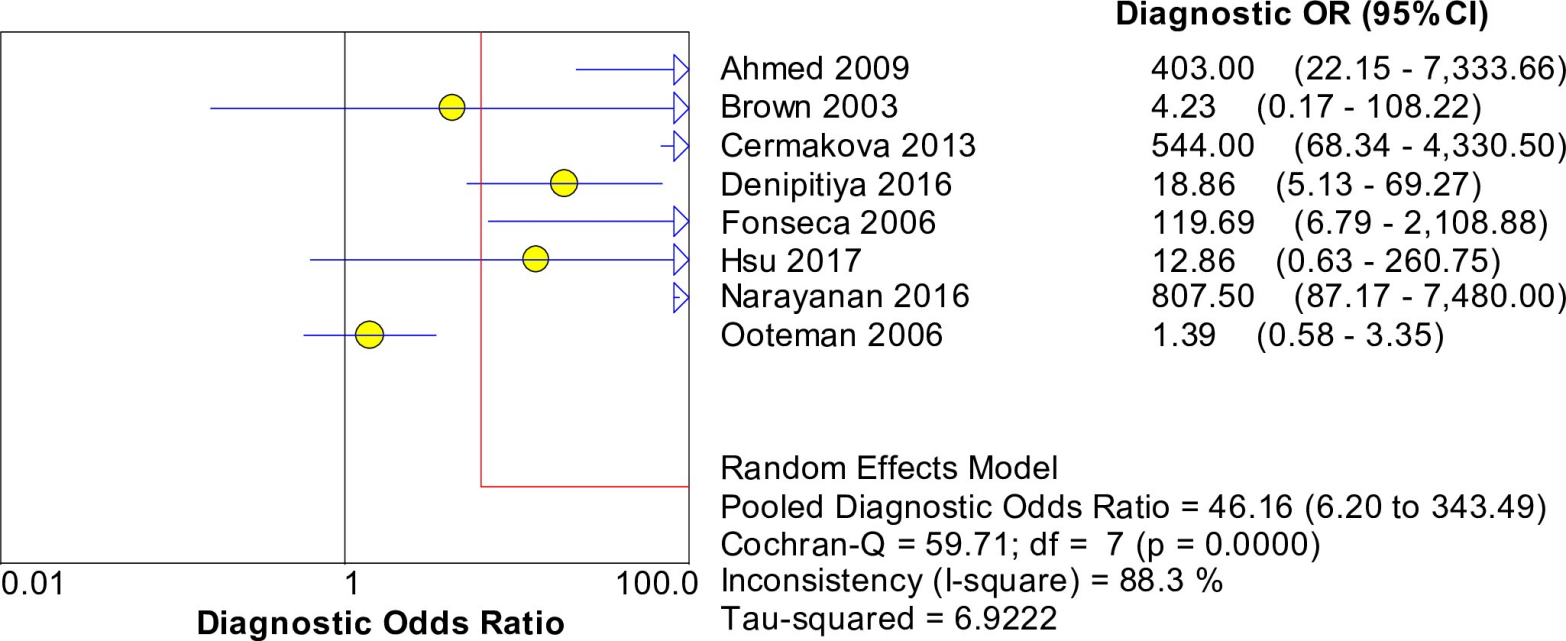
Forest plot of DOR of targeting the *secY* gene in the detection of *Leptospira*.

**Fig 10:**
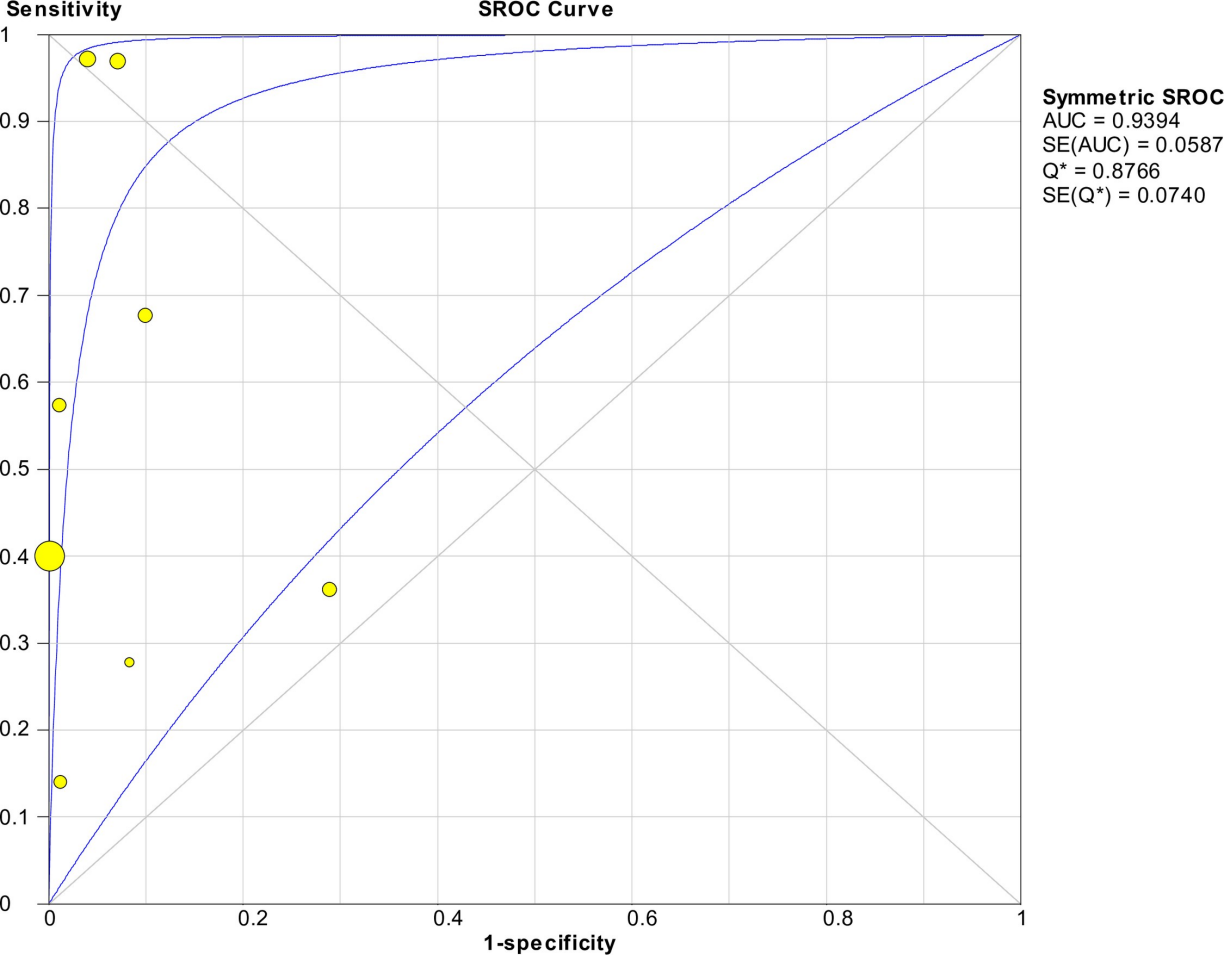
SROC curve of targeting the *secY* gene in the detection of *Leptospira*.

Fig 8 shows the forest plots of the sensitivities and specificities of techniques targeting the *secY* gene. The pooled sensitivity and specificity were 0.56 (95% CI: 0.50–0.63) and 0.98 (95% CI: 0.97–0.98), respectively. The pooled DOR was valued at 46.16 (95% CI: 6.20–343.49) and is presented in Fig 9. Fig 10 shows the SROC curve with the AUC at 0.94 and the pooled diagnostic accuracy (Q*) at 0.88. Among these studies, significant heterogeneity was observed (sensitivity, *I*^2^ = 92.0%; specificity, *I*^2^ = 92.4%; DOR, *I*^2^ = 88.3%).

The Spearman correlation coefficient was 0.000 with *p* = 1.000 (> 0.05), indicating that the heterogeneity was not due to the threshold effect. Thus, meta-regression and subgroup analyses were performed to explore the source of heterogeneity. The meta-regression analysis did not demonstrate that the two covariates contributed to the heterogeneity (*p* > 0.05 for both covariates). Subgroup analyses based on these covariates were performed, and the results are shown in Table 4. The AUC for the subgroup of index test (qPCR) was not determined because of the low number of studies (n = 2). Analysis for animal samples was not performed because only one study was present in this category.

**Table 4:** Meta-analysis results of studies targeting the *secY* gene for the detection of *Leptospira*.

	Sensitivity (95% CI)	Specificity (95% CI)	PLR (95% CI)	NLR (95% CI)	DOR (95% CI)	AUC
**All studies**	0.56 (0.50–0.63)	0.98 (0.97–0.98)	12.94 (3.74–44.72)	0.49 (0.30–0.82)	46.16 (6.20–343.49)	0.94
**Subgroup (Type of index test)**						
**Conventional PCR**	0.49 (0.41–0.56)	0.98 (0.97–0.99)	15.63 (2.04–119.78)	0.60 (0.37–0.97)	39.57 (2.44–642.05)	0.91
**qPCR**	0.74 (0.63–0.83)	0.93 (0.87–0.96)	10.50 (4.65–23.71)	0.13 (0.00–6.15)	65.78 (3.37–1284.84)	NA
**Subgroup (Type of index test)**						
**Human samples**	0.61 (0.54–0.67)	0.97 (0.96–0.98)	13.17 (3.52–49.33)	0.45 (0.27–0.77)	54.70 (5.97–501.49)	0.94

### Accuracy of nucleic acid techniques targeting the *flaB* gene

**Fig 11:**
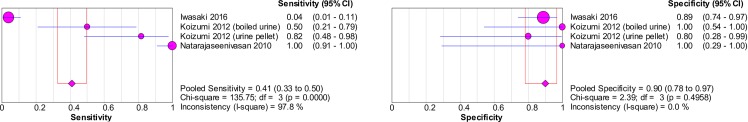
Forest plots of sensitivity and specificity of studies using *flaB* as the target gene for the detection of *Leptospira*.

**Fig 12:**
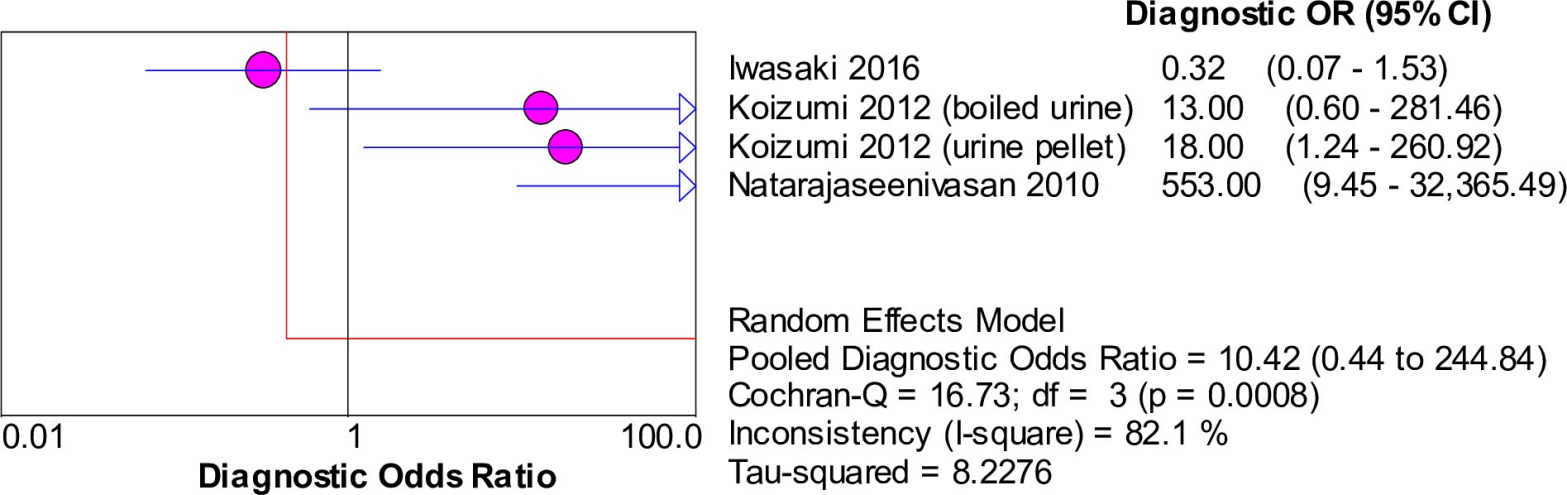
Forest plot of DOR of targeting the *flaB* gene in the detection of *Leptospira*.

**Fig 13:**
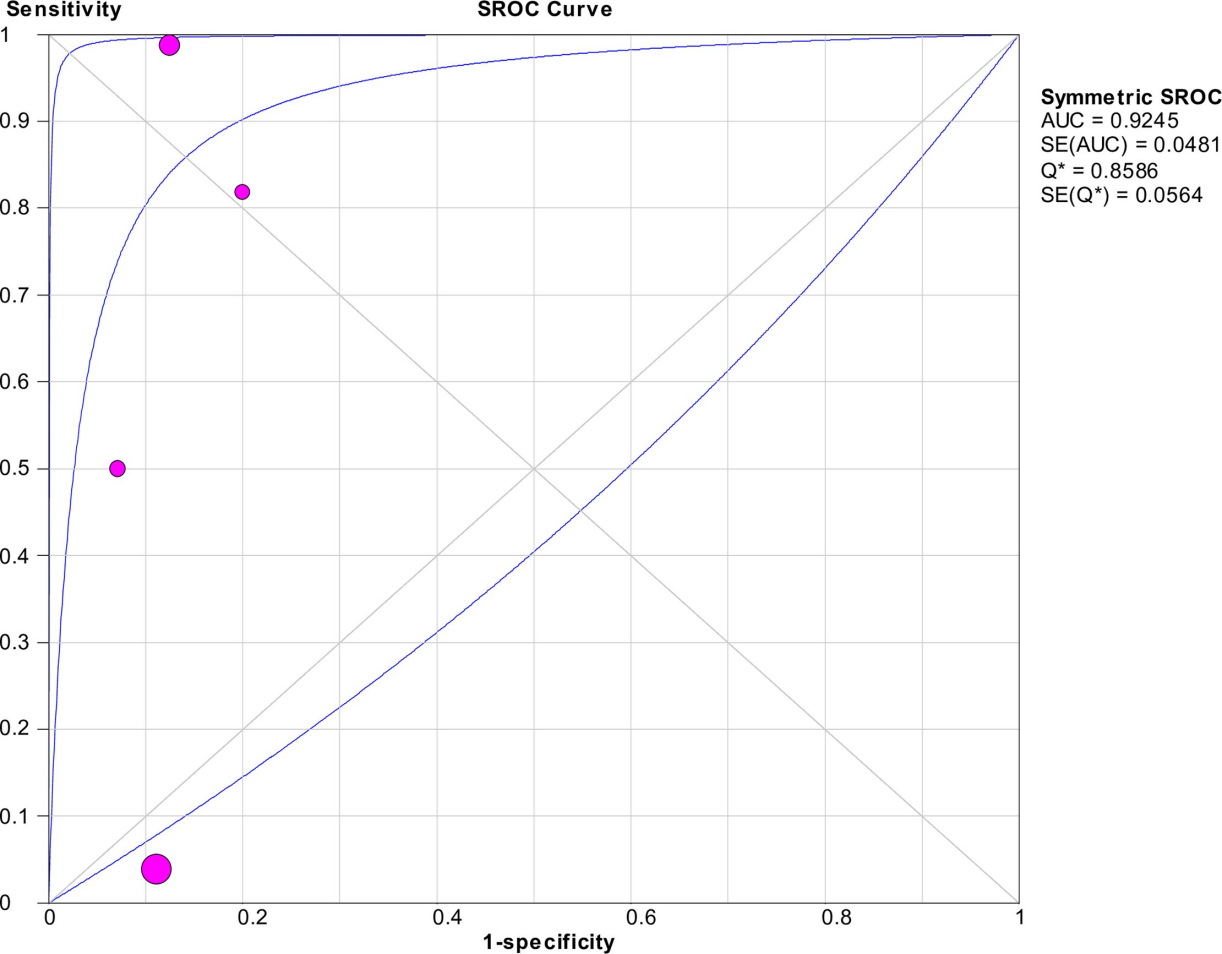
SROC curve of targeting the *flaB* gene in the detection of *Leptospira*.

For techniques targeting the *flaB* gene, the pooled sensitivity and specificity of the included studies were 0.41 (95% CI: 0.33–0.50) and 0.90 (95% CI: 0.78–0.97), respectively. The forest plots of the sensitivities and specificities are shown in Fig 11. The pooled DOR was 10.42 (95% CI: 0.44–244.84), as shown in Fig 12. The SROC curve is presented in Fig 13, with the AUC at 0.92 and pooled diagnostic accuracy (Q*) at 0.86. Significant heterogeneity was observed when computing the pooled sensitivity (*I*^2^ = 97.8%) and DOR (*I*^2^ = 82.1%).

The Spearman correlation coefficient was 0.600 with *p* = 0.400 (> 0.05), eliminating the possibility of the threshold effect. The meta-regression analysis did not demonstrate that the two covariates contributed to the heterogeneity (*p* > 0.05 for both covariates). Subgroup analyses were not performed because of the limited number of studies in each subgroup.

## Discussion

In this meta-analysis, 42 studies involving 7414 samples were included to investigate the diagnostic accuracy of various nucleic acid techniques. Many nucleic acid diagnostics for leptospirosis have been developed and designed to either target housekeeping genes that are common to all species of *Leptospira* or pathogenic species-specific genes. Here, from the literature searched, we pooled and analyzed the diagnostic performance of nucleic acid assays targeting the *rrs*, *lipL32*, *secY* and *flaB* genes. The IS1500, LP1, *lfb1*, and *lipL41* genes were not pooled and analyzed because only one study included each of these genes [12, 24, 28, 39].

The *rrs* gene is a housekeeping gene found ubiquitously among leptospires. The present meta-analysis showed that assays targeting the *rrs* gene have been well-established and largely used for diagnostics. Targeting the *rrs* gene can allow the detection of pathogenic or saprophytic *Leptospira* species [59]. The *rrs* gene is also present in two copies per *Leptospira*, which could consequently increase its chance of being amplified [61, 62]. Meanwhile, the *secY* gene encodes for preprotein translocase for *Leptospira* and is located within the S10-spc-α locus containing genes for ribosomal proteins [63]. Similar to the *rrs* gene, it is a housekeeping gene that is also common to all leptospires [11]. The *secY* gene consists of alternating conserved and variable regions, making it suitable to design primers that can generate amplicons across the *Leptospira* genus and enable phylogenetic interpretation through the variable regions [64].

The *lipL32* gene encodes a major lipoprotein located in the outer membrane of leptospires. The *lipL32* gene is present in all species from both pathogenic and intermediate strains but absent in saprophytic strains, suggesting its critical role in infection [65, 66]. The sequence of the *lipL32* gene is highly conserved across the pathogenic species of *Leptospira*, with more than 94% amino acid sequence identities [67]. Thus, the absence of *lipL32* in saprophytic *Leptospira* makes it a specific and appropriate gene target for diagnosing leptospirosis [25]. Another gene that can be used to differentiate between pathogenic and saprophytic leptospires is the *flaB* gene. This gene encodes for flagellin, a class B polypeptide subunit of the periplasmic flagella. The sequence of *flaB* is also highly conserved among pathogenic serovars of *Leptospira* [68]. Similar to the *lipL32* gene, the absence of *flaB* in the saprophytic strains allows this gene to be a good target for detecting pathogenic leptospires [50, 69].

As shown in Table 5, nucleic acid techniques targeting the *secY* gene exhibited better pooled sensitivity and specificity when compared against assays targeting the *rrs*, *lipL32*, or *flaB* gene. Sensitivity and specificity are true performance statistics of the test, where sensitivity measures for the proportion of samples tested positive among those tested positive using a reference test while specificity measures the proportion of samples tested negative among those tested negative in a reference test [70]. The PLR of assays targeting *secY* is also the highest among others and is the only one with a value more than 10. This result indicates that the positive results obtained from assays targeting *secY* are useful for the confirmation of leptospirosis [71]. Sensitivity and specificity play important roles in determining the DOR of a test. For example, tests with high sensitivity and specificity with low FPs and FNs result in a high DOR [16]. In our analysis, the pooled DOR of assays targeting *secY* was the highest among other genes. Collectively, the pooled sensitivity, specificity, DOR, likelihood ratio, and AUC data all support that assays targeting the *secY* gene are highly discriminatory for the detection of *Leptospira*.

**Table 5:** Summary of diagnostic accuracy measures of genetic markers for the detection of *Leptospira* in clinical samples.

Gene	Sensitivity (95% CI)	Specificity (95% CI)	PLR (95% CI)	NLR (95% CI)	DOR (95% CI)	AUC
***rrs***	0.51 (0.48–0.54)	0.90 (0.89–0.91)	4.36 (2.80–6.79)	0.56 (0.44–0.70)	13.58 (6.66–27.67)	0.88
***lipL32***	0.42 (0.39–0.46)	0.95 (0.94–0.97)	9.42 (5.64–15.75)	0.61 (0.51–0.71)	19.71 (10.15–38.29)	0.92
***secY***	0.56 (0.50–0.63)	0.98 (0.97–0.98)	12.94 (3.74–44.72)	0.49 (0.30–0.82)	46.16 (6.20–343.49)	0.94
***flaB***	0.41 (0.33–0.50)	0.90 (0.78–0.97)	2.43 (0.44–13.52)	0.36 (0.11–1.18)	10.42 (0.44–244.84)	0.92

Stratified analyses were performed for each gene analyzed according to the type of index test. For the *rrs* gene, subgroup analysis revealed that nested PCR assays targeting the gene are superior over other tests and slightly better than qPCR, as exhibited by the higher DOR and AUC of the SROC curve. In a previous study, qRT-PCR assay targeting the *rrs* gene was compared against nested PCR assay of the same gene, and the diagnostic performance was comparable between the two [72]. Another study on the detection of *Strongyloides stercoralis* also found that nested PCR shows better diagnostic sensitivity than real-time PCR [73]. However superior nested PCR is, real-time PCR methods are usually preferred over the former because they provide an accurate diagnosis faster than nested PCR assays [74]. By contrast, qPCR assay targeting the *lipL32* gene showed slightly better performance than nested PCR assay, but this finding is inconclusive because AUC data for nested PCR assay was not computed due to the lack of studies. As for the *secY* gene, although AUC data for qPCR were not determined, the sensitivity and DOR of qPCR were significantly higher than those of conventional PCR. When the data were stratified according to the type of samples, assays targeting the *rrs* gene showed slightly better diagnostic performance on animal than human samples. Meanwhile, the diagnostic performance of the other genes on animal samples was not computed because of the lack of studies. As for human samples, assays targeting the *secY* gene showed the best diagnostic performance in terms of DOR, followed by those targeting *lipL32*, *rrs*, and *flaB*.

However, the findings of this meta-analysis should be interpreted with caution, considering the significant unexplained heterogeneity. The heterogeneity of the analyzed studies could be due to the vastly different sample sizes among the included studies. Moreover, the low number of studies in assays targeting the *flaB* gene and in some subgroups might have also biased the results. Another possible contribution to the heterogeneity of the studies was the variability in the DNA sample extraction approach, reference test, and the stage of the disease when samples were collected from the patients and animals. The timing of sample collection is crucial for the detection of *Leptospira* DNA because it is present in the blood of the patient in the first 5 to 10 days after the onset of the disease [11]. As mentioned previously, although MAT has been the gold standard for the diagnosis of leptospirosis, its application is limited by its difficulty to be standardized [8], suggesting that this limitation also contributed to the heterogeneity observed. In these nucleic acid assays, the different targeting regions within each gene would represent a major factor influencing the sensitivity and specificity of a diagnostic test. These limitations could have negatively influenced the overall results of this work.

In short, current evidence suggests that the *secY* gene has better diagnostic accuracy measures with *lipL32*, *flaB*, and *rrs* coming close as promising genetic markers for leptospirosis diagnosis. However, the high degree of heterogeneity observed in this meta-analysis mitigates any conclusions drawn from the combined data. Nevertheless, future studies evaluating the nucleic acid-based diagnostic assays should consider the timing and stage of the disease for sample collection, the choice of reference test to be compared with, or the statistical methods to optimize the imperfect reference tests, in an effort to reduce the heterogeneity between the studies while increasing the comparability of results.

## Supporting information

S1 Checklist PRISMA checklist. (DOC)

## References

[1] Levett PN. Leptospirosis. Clin Microbiol Rev. 2001;14(2):296–326. 10.1128/CMR.14.2.296-326.2001 11292640 PMC88975

[2] Costa F, Hagan JE, Calcagno J, Kane M, Torgerson P, Martinez-Silveira MS, et al Global Morbidity and Mortality of Leptospirosis: A Systematic Review. PLoS Negl Trop Dis. 2015;9(9):e0003898 10.1371/journal.pntd.0003898 26379143 PMC4574773

[3] McBride AJ, Athanazio DA, Reis MG, Ko AI. Leptospirosis. Curr Opin Infect Dis. 2005;18(5):376–86. 10.1097/01.qco.0000178824.05715.2c 16148523

[4] Bharti AR, Nally JE, Ricaldi JN, Matthias MA, Diaz MM, Lovett MA, et al Leptospirosis: a zoonotic disease of global importance. Lancet Infect Dis. 2003;3(12):757–71. 10.1016/s1473-3099(03)00830-2 14652202

[5] Lau CL, Smythe LD, Craig SB, Weinstein P. Climate change, flooding, urbanisation and leptospirosis: fuelling the fire? Trans R Soc Trop Med Hyg. 2010;104(10):631–8. 10.1016/j.trstmh.2010.07.002 20813388

[6] Sayyed Mousavi MN, Sadeghi J, Aghazadeh M, Asgharzadeh M, Samadi Kafil H. Current advances in urban leptospirosis diagnosis. Rev Med Microbiol. 2017;28(3):119–23.

[7] Goris MG, Hartskeerl RA. Leptospirosis serodiagnosis by the microscopic agglutination test. Curr Protoc Microbiol. 2014;32:Unit 12E 5. 10.1002/9780471729259.mc12e05s32 24510846

[8] Musso D, La Scola B. Laboratory diagnosis of leptospirosis: a challenge. J Microbiol Immunol Infect. 2013;46(4):245–52. 10.1016/j.jmii.2013.03.001 23639380

[9] Picardeau M. Diagnosis and epidemiology of leptospirosis. Med Mal Infect. 2013;43(1):1–9. 10.1016/j.medmal.2012.11.005 23337900

[10] Waggoner JJ, Pinsky BA. Molecular diagnostics for human leptospirosis. Curr Opin Infect Dis. 2016;29(5):440–5. 10.1097/QCO.0000000000000295 27537829 PMC5127924

[11] Ahmed A, Engelberts MF, Boer KR, Ahmed N, Hartskeerl RA. Development and validation of a real-time PCR for detection of pathogenic leptospira species in clinical materials. PLoS One. 2009;4(9):e7093 10.1371/journal.pone.0007093 19763264 PMC2740861

[12] Merien F, Portnoi D, Bourhy P, Charavay F, Berlioz-Arthaud A, Baranton G. A rapid and quantitative method for the detection of Leptospira species in human leptospirosis. FEMS Microbiol Lett. 2005;249(1):139–47. 10.1016/j.femsle.2005.06.011 16006065

[13] Palaniappan RU, Chang YF, Chang CF, Pan MJ, Yang CW, Harpending P, et al Evaluation of lig-based conventional and real time PCR for the detection of pathogenic leptospires. Mol Cell Probes. 2005;19(2):111–7. 10.1016/j.mcp.2004.10.002 15680212

[14] Smythe LD, Smith IL, Smith GA, Dohnt MF, Symonds ML, Barnett LJ, et al A quantitative PCR (TaqMan) assay for pathogenic Leptospira spp. BMC Infect Dis. 2002;2:13 10.1186/1471-2334-2-13 12100734 PMC117785

[15] Toyokawa T, Ohnishi M, Koizumi N. Diagnosis of acute leptospirosis. Expert Rev Anti Infect Ther. 2011;9(1):111–21. 10.1586/eri.10.151 21171882

[16] Glas AS, Lijmer JG, Prins MH, Bonsel GJ, Bossuyt PM. The diagnostic odds ratio: a single indicator of test performance. J Clin Epidemiol. 2003;56(11):1129–35. 10.1016/s0895-4356(03)00177-x 14615004

[17] DerSimonian R, Laird N. Meta-analysis in clinical trials. Control Clin Trials. 1986;7(3):177–88. 10.1016/0197-2456(86)90046-2 3802833

[18] Walter SD. Properties of the summary receiver operating characteristic (SROC) curve for diagnostic test data. Stat Med. 2002;21(9):1237–56. 10.1002/sim.1099 12111876

[19] Higgins JP, Thompson SG, Deeks JJ, Altman DG. Measuring inconsistency in meta-analyses. BMJ. 2003;327(7414):557–60. 10.1136/bmj.327.7414.557 12958120 PMC192859

[20] Devillé WL, Buntinx F, Bouter LM, Montori VM, de Vet HCW, van der Windt DAWM, et al Conducting systematic reviews of diagnostic studies: didactic guidelines. BMC Med Res Methodol. 2002;2(1):9. 12097142 10.1186/1471-2288-2-9 PMC117243

[21] Zamora J, Abraira V, Muriel A, Khan K, Coomarasamy A. Meta-DiSc: a software for meta-analysis of test accuracy data. BMC Med Res Methodol. 2006;6:31 10.1186/1471-2288-6-31 16836745 PMC1552081

[22] Agampodi SB, Matthias MA, Moreno AC, Vinetz JM. Utility of quantitative polymerase chain reaction in leptospirosis diagnosis: association of level of leptospiremia and clinical manifestations in Sri Lanka. Clin Infect Dis. 2012;54(9):1249–55. 10.1093/cid/cis035 22354922 PMC3404689

[23] Blanco RM, Romero EC. Evaluation of nested polymerase chain reaction for the early detection of Leptospira spp. DNA in serum samples from patients with leptospirosis. Diagn Microbiol Infect Dis. 2014;78(4):343–6. 10.1016/j.diagmicrobio.2013.12.009 24445157

[24] Fonseca Cde A, Teixeira MM, Romero EC, Tengan FM, Silva MV, Shikanai-Yasuda MA. Leptospira DNA detection for the diagnosis of human leptospirosis. J Infect. 2006;52(1):15–22. 10.1016/j.jinf.2005.02.022 16368457

[25] Hsu YH, Chou SJ, Chang CC, Pan MJ, Yang WC, Lin CF, et al Development and validation of a new loop-mediated isothermal amplification for detection of pathogenic Leptospira species in clinical materials. J Microbiol Methods. 2017;141:55–9. 10.1016/j.mimet.2017.07.010 28756184

[26] Iwasaki H, Chagan-Yasutan H, Leano PS, Koizumi N, Nakajima C, Taurustiati D, et al Combined antibody and DNA detection for early diagnosis of leptospirosis after a disaster. Diagn Microbiol Infect Dis. 2016;84(4):287–91. 10.1016/j.diagmicrobio.2016.01.001 26860351

[27] Koizumi N, Nakajima C, Harunari T, Tanikawa T, Tokiwa T, Uchimura E, et al A new loop-mediated isothermal amplification method for rapid, simple, and sensitive detection of Leptospira spp. in urine. J Clin Microbiol. 2012;50(6):2072–4. 10.1128/JCM.00481-12 22422858 PMC3372145

[28] Sonthayanon P, Chierakul W, Wuthiekanun V, Thaipadungpanit J, Kalambaheti T, Boonsilp S, et al Accuracy of loop-mediated isothermal amplification for diagnosis of human leptospirosis in Thailand. Am J Trop Med Hyg. 2011;84(4):614–20. 10.4269/ajtmh.2011.10-0473 21460019 PMC3062458

[29] Riediger IN, Stoddard RA, Ribeiro GS, Nakatani SM, Moreira SDR, Skraba I, et al Rapid, actionable diagnosis of urban epidemic leptospirosis using a pathogenic Leptospira lipL32-based real-time PCR assay. PLoS Negl Trop Dis. 2017;11(9):e0005940 10.1371/journal.pntd.0005940 28915243 PMC5617227

[30] Thaipadungpanit J, Chierakul W, Wuthiekanun V, Limmathurotsakul D, Amornchai P, Boonslip S, et al Diagnostic accuracy of real-time PCR assays targeting 16S rRNA and lipL32 genes for human leptospirosis in Thailand: a case-control study. PLoS One. 2011;6(1):e16236 10.1371/journal.pone.0016236 21283633 PMC3026019

[31] Vanasco NB, Jacob P, Landolt N, Chiani Y, Schmeling MF, Cudos C, et al Diagnostic accuracy of an IgM enzyme-linked immunosorbent assay and comparison with 2 polymerase chain reactions for early diagnosis of human leptospirosis. Diagn Microbiol Infect Dis. 2016;84(4):292–7. 10.1016/j.diagmicrobio.2016.01.002 26867967

[32] Villumsen S, Pedersen R, Borre MB, Ahrens P, Jensen JS, Krogfelt KA. Novel TaqMan(R) PCR for detection of Leptospira species in urine and blood: pit-falls of in silico validation. J Microbiol Methods. 2012;91(1):184–90. 10.1016/j.mimet.2012.06.009 22750039

[33] Agampodi SB, Dahanayaka NJ, Nockler K, Mayer-Scholl A, Vinetz JM. Redefining Gold Standard Testing for Diagnosing Leptospirosis: Further Evidence from a Well-Characterized, Flood-Related Outbreak in Sri Lanka. Am J Trop Med Hyg. 2016;95(3):531–6. 10.4269/ajtmh.16-0033 27402521 PMC5014254

[34] Ahmed A, van der Linden H, Hartskeerl RA. Development of a recombinase polymerase amplification assay for the detection of pathogenic Leptospira. Int J Environ Res Public Health. 2014;11(5):4953–64. 10.3390/ijerph110504953 24814943 PMC4053868

[35] Backstedt BT, Buyuktanir O, Lindow J, Wunder EA Jr., Reis MG, Usmani-Brown S, et al Efficient Detection of Pathogenic Leptospires Using 16S Ribosomal RNA. PLoS One. 2015;10(6):e0128913 10.1371/journal.pone.0128913 26091292 PMC4474562

[36] Bandara K, Weerasekera MM, Gunasekara C, Ranasinghe N, Marasinghe C, Fernando N. Utility of modified Faine's criteria in diagnosis of leptospirosis. BMC Infect Dis. 2016;16(1):446 10.1186/s12879-016-1791-9 27554098 PMC4995749

[37] Boonsilp S, Thaipadungpanit J, Amornchai P, Wuthiekanun V, Chierakul W, Limmathurotsakul D, et al Molecular detection and speciation of pathogenic Leptospira spp. in blood from patients with culture-negative leptospirosis. BMC Infect Dis. 2011;11(1):338. 22151687 10.1186/1471-2334-11-338 PMC3297668

[38] Brown PD, Carrington DG, Gravekamp C, van de Kemp H, Edwards CN, Jones SR, et al Direct detection of leptospiral material in human postmortem samples. Res Microbiol. 2003;154(8):581–6. 10.1016/S0923-2508(03)00166-9 14527659

[39] Cameron CE, Zuerner RL, Raverty S, Colegrove KM, Norman SA, Lambourn DM, et al Detection of pathogenic Leptospira bacteria in pinniped populations via PCR and identification of a source of transmission for zoonotic leptospirosis in the marine environment. J Clin Microbiol. 2008;46(5):1728–33. 10.1128/JCM.02022-07 18367568 PMC2395076

[40] Cermakova Z, Kucerova P, Valenta Z, Pliskova L, Bolehovska R, Prasil P, et al Leptospirosis: possibilities of early laboratory and clinical diagnosis. Central European Journal of Medicine. 2013;8(1):84–9.

[41] Denipitiya DT, Chandrasekharan NV, Abeyewickreme W, Hartskeerl CM, Hartskeerl RA, Jiffrey AM, et al Application of a real time Polymerase Chain Reaction (PCR) assay for the early diagnosis of human leptospirosis in Sri Lanka. Biologicals. 2016;44(6):497–502. 10.1016/j.biologicals.2016.09.004 27707560

[42] Gonzalez S, Geymonat JP, Hernandez E, Marques JM, Schelotto F, Varela G. Usefulness of real-time PCR assay targeting lipL32 gene for diagnosis of human leptospirosis in Uruguay. J Infect Dev Ctries. 2013;7(12):941–5. 10.3855/jidc.4110 24334940

[43] Gokmen TG, Soyal A, Kalayci Y, Onlen C, Koksal F. Comparison of 16S rRNA-PCR-RFLP, LipL32-PCR and OmpL1-PCR methods in the diagnosis of leptospirosis. Rev Inst Med Trop Sao Paulo. 2016;58(0):64. 27680169 10.1590/S1678-9946201658064 PMC5048635

[44] Lakshmipriya C, Anandhagiri S, Natarajaseenivasan K. Prevalence of canine leptospirosis in Tiruchirappalli, Tamil Nadu. Indian J Anim Sci. 2012;82(7):702–5.

[45] McAvin JC, Kengluecha A, Takhampunya R, Richardson JH. A field-expedient method for detection of leptospirosis causative agents in rodents. US Army Med Dep J. 2012:22–8. 22815161

[46] Merien F, Amouriaux P, Perolat P, Baranton G, Saint Girons I. Polymerase chain reaction for detection of Leptospira spp. in clinical samples. J Clin Microbiol. 1992;30(9):2219–24. 1400983 10.1128/jcm.30.9.2219-2224.1992 PMC265482

[47] Mohd Ali MR, Mohd Safee AW, Ismail NH, Abu Sapian R, Mat Hussin H, Ismail N, et al Development and validation of pan-Leptospira Taqman qPCR for the detection of Leptospira spp. in clinical specimens. Mol Cell Probes. 2018;38:1–6. 10.1016/j.mcp.2018.03.001 29524642

[48] Mullan S, Panwala TH. Polymerase Chain Reaction: An Important Tool for Early Diagnosis of Leptospirosis Cases. J Clin Diagn Res. 2016;10(12):DC08–DC11. 10.7860/JCDR/2016/22462.9010 28208854 PMC5296427

[49] Narayanan R, Sumathi G, Prabhakaran SG, Shanmughapriya S, Natarajaseenivasan K. Paediatric leptospirosis: A population based case-control study from Chennai, India. Indian J Med Microbiol. 2016;34(2):228–32. 10.4103/0255-0857.180353 27080780

[50] Natarajaseenivasan K, Vijayachari P, Sharma S, Sugunan AP, Vedhagiri K, Selvin J, et al FlaB PCR-based identification of pathogenic leptospiral isolates. J Microbiol Immunol Infect. 2010;43(1):62–9. 10.1016/S1684-1182(10)60009-6 20434125

[51] Ooteman MC, Vago AR, Koury MC. Evaluation of MAT, IgM ELISA and PCR methods for the diagnosis of human leptospirosis. J Microbiol Methods. 2006;65(2):247–57. 10.1016/j.mimet.2005.07.015 16253361

[52] Ram S, Vimalin JM, Jambulingam M, Tiru V, Gopalakrishnan RK, Naraharirao MH. Application of PCR-based DNA sequencing technique for the detection of Leptospira in peripheral blood of septicemia patients. Malays J Microbiol. 2012;8(1):26–33.

[53] Reller ME, Wunder EA Jr., Miles JJ, Flom JE, Mayorga O, Woods CW, et al Unsuspected leptospirosis is a cause of acute febrile illness in Nicaragua. PLoS Negl Trop Dis. 2014;8(7):e2941 10.1371/journal.pntd.0002941 25058149 PMC4109853

[54] Romero EC, Blanco RM, Yasuda PH. Aseptic meningitis caused by Leptospira spp diagnosed by polymerase chain reaction. Mem Inst Oswaldo Cruz. 2010;105(8):988–92. 10.1590/s0074-02762010000800007 21225195

[55] Shekatkar S, Harish BN, Parija SC. Diagnosis of leptospirosis by polymerase chain reaction. Int J Pharma Bio Sci. 2010;1(3):1–6.

[56] Sivasankari K, Shanmughapriya S, Natarajaseenivasan K. Leptospiral renal colonization status in asymptomatic rural population of Tiruchirapalli district, Tamilnadu, India. Pathog Glob Health. 2016;110(4–5):209–15. 10.1080/20477724.2016.1222054 27549577 PMC5072110

[57] Slack A, Symonds M, Dohnt M, Harris C, Brookes D, Smythe L. Evaluation of a modified Taqman assay detecting pathogenic Leptospira spp. against culture and Leptospira-specific IgM enzyme-linked immunosorbent assay in a clinical environment. Diagn Microbiol Infect Dis. 2007;57(4):361–6. 10.1016/j.diagmicrobio.2006.10.004 17188447

[58] Tulsiani SM, Graham GC, Dohnt MF, Burns MA, Craig SB. Maximizing the chances of detecting pathogenic leptospires in mammals: the evaluation of field samples and a multi-sample-per-mammal, multi-test approach. Ann Trop Med Parasitol. 2011;105(2):145–62. 10.1179/136485911X12899838683205 21396251 PMC4084665

[59] Waggoner JJ, Balassiano I, Abeynayake J, Sahoo MK, Mohamed-Hadley A, Liu Y, et al Sensitive real-time PCR detection of pathogenic Leptospira spp. and a comparison of nucleic acid amplification methods for the diagnosis of leptospirosis. PLoS One. 2014;9(11):e112356 10.1371/journal.pone.0112356 25379890 PMC4224423

[60] Woods K, Nic-Fhogartaigh C, Arnold C, Boutthasavong L, Phuklia W, Lim C, et al A comparison of two molecular methods for diagnosing leptospirosis from three different sample types in patients presenting with fever in Laos. Clin Microbiol Infect. 2018;24(9):1017 e1–e7. 29092789 10.1016/j.cmi.2017.10.017 PMC6125144

[61] Nascimento AL, Verjovski-Almeida S, Van Sluys MA, Monteiro-Vitorello CB, Camargo LE, Digiampietri LA, et al Genome features of Leptospira interrogans serovar Copenhageni. Braz J Med Biol Res. 2004;37(4):459–77. 10.1590/s0100-879x2004000400003 15064809 PMC2666282

[62] Picardeau M, Bulach DM, Bouchier C, Zuerner RL, Zidane N, Wilson PJ, et al Genome sequence of the saprophyte Leptospira biflexa provides insights into the evolution of Leptospira and the pathogenesis of leptospirosis. PLoS One. 2008;3(2):e1607 10.1371/journal.pone.0001607 18270594 PMC2229662

[63] Zuerner RL, Hartskeerl RA, van de Kemp H, Bal AE. Characterization of the Leptospira interrogans S10-spc-alpha operon. FEMS Microbiol Lett. 2000;182(2):303–8. 10.1111/j.1574-6968.2000.tb08912.x 10620683

[64] Victoria B, Ahmed A, Zuerner RL, Ahmed N, Bulach DM, Quinteiro J, et al Conservation of the S10-spc-alpha locus within otherwise highly plastic genomes provides phylogenetic insight into the genus Leptospira. PLoS One. 2008;3(7):e2752 10.1371/journal.pone.0002752 18648538 PMC2481283

[65] Fouts DE, Matthias MA, Adhikarla H, Adler B, Amorim-Santos L, Berg DE, et al What Makes a Bacterial Species Pathogenic?:Comparative Genomic Analysis of the Genus Leptospira. PLoS Negl Trop Dis. 2016;10(2):e0004403 10.1371/journal.pntd.0004403 26890609 PMC4758666

[66] Thibeaux R, Girault D, Bierque E, Soupe-Gilbert ME, Rettinger A, Douyere A, et al Biodiversity of Environmental Leptospira: Improving Identification and Revisiting the Diagnosis. Front Microbiol. 2018;9:816 10.3389/fmicb.2018.00816 29765361 PMC5938396

[67] Pinne M, Haake DA. LipL32 Is a Subsurface Lipoprotein of Leptospira interrogans: presentation of new data and reevaluation of previous studies. PLoS One. 2013;8(1):e51025 10.1371/journal.pone.0051025 23323152 PMC3544172

[68] Lin M, Surujballi O, Nielsen K, Nadin-Davis S, Randall G. Identification of a 35-Kilodalton Serovar-Cross-Reactive Flagellar Protein, FlaB, from Leptospira interrogans by N-Terminal Sequencing, Gene Cloning, and Sequence Analysis. Infect Immun. 1997;65(10):4355–9. 9317049 10.1128/iai.65.10.4355-4359.1997 PMC175625

[69] Kawabata H, Dancel LA, Villanueva SY, Yanagihara Y, Koizumi N, Watanabe H. flaB-polymerase chain reaction (flaB-PCR) and its restriction fragment length polymorphism (RFLP) analysis are an efficient tool for detection and identification of Leptospira spp. Microbiol Immunol. 2001;45(6):491–6. 10.1111/j.1348-0421.2001.tb02649.x 11497225

[70] Leeflang MM. Systematic reviews and meta-analyses of diagnostic test accuracy. Clin Microbiol Infect. 2014;20(2):105–13. 10.1111/1469-0691.12474 24274632

[71] McGee S. Simplifying likelihood ratios. J Gen Intern Med. 2002;17(8):646–9. 10.1046/j.1525-1497.2002.10750.x 12213147 PMC1495095

[72] Esteves LM, Bulhoes SM, Branco CC, Carreira T, Vieira ML, Gomes-Solecki M, et al Diagnosis of Human Leptospirosis in a Clinical Setting: Real-Time PCR High Resolution Melting Analysis for Detection of Leptospira at the Onset of Disease. Sci Rep. 2018;8(1):9213 10.1038/s41598-018-27555-2 29907838 PMC6003994

[73] Sharifdini M, Mirhendi H, Ashrafi K, Hosseini M, Mohebali M, Khodadadi H, et al Comparison of Nested Polymerase Chain Reaction and Real-Time Polymerase Chain Reaction with Parasitological Methods for Detection of Strongyloides stercoralis in Human Fecal Samples. Am J Trop Med Hyg. 2015;93(6):1285–91. 10.4269/ajtmh.15-0309 26350449 PMC4674247

[74] Espy MJ, Uhl JR, Sloan LM, Buckwalter SP, Jones MF, Vetter EA, et al Real-time PCR in clinical microbiology: applications for routine laboratory testing. Clin Microbiol Rev. 2006;19(1):165–256. 10.1128/CMR.19.1.165-256.2006 16418529 PMC1360278

